# Comparison of the structures and topologies of plasma extracted circulating nuclear and mitochondrial cell-free DNA

**DOI:** 10.3389/fgene.2023.1104732

**Published:** 2023-04-20

**Authors:** Ekaterina Pisareva, Benoit Roch, Cynthia Sanchez, Brice Pastor, Alexia Mirandola, Mona Diab-Assaf, Thibault Mazard, Corinne Prévostel, Zahra Al Amir Dache, Alain R. Thierry

**Affiliations:** ^1^ IRCM, Institut de Recherche en Cancérologie de Montpellier, INSERM U1194, Montpellier University, Montpellier, France; ^2^ Thoracic Oncology Unit, Arnaud De Villeneuve Hospital, University Hospital of Montpellier, Montpellier, France; ^3^ Faculty of Sciences II, Lebanese University Fanar, Beirut, Lebanon; ^4^ ICM, Institut Régional du Cancer de Montpellier, Montpellier, France

**Keywords:** circulating DNA, mitochondria, whole genome sequencing, Q-PCR, diagnostics, structure, topology, extracellular vesicles

## Abstract

**Introduction:** The function, origin and structural features of circulating nuclear DNA (cir-nDNA) and mitochondrial DNA (cir-mtDNA) are poorly known, even though they have been investigated in numerous clinical studies, and are involved in a number of routine clinical applications. Based on our previous report disproving the conventional plasma isolation used for cirDNA analysis, this work enables a direct topological comparison of the circulating structures associated with nuclear DNA and mitochondrial cell-free DNA.

**Materials and methods:** We used a Q-PCR and low-pass whole genome sequencing (LP-WGS) combination approach of cir-nDNA and cir-mtDNA, extracted using a procedure that eliminates platelet activation during the plasma isolation process to prevent mitochondria release in the extracellular milieu. Various physical procedures, such as filtration and differential centrifugation, were employed to infer their circulating structures.

**Results:** DSP-S cir-mtDNA mean size profiles distributed on a slightly shorter range than SSP-S. SSP-S detected 40-fold more low-sized cir-mtDNA fragments (<90 bp/nt) and three-fold less long-sized fragments (>200 bp/nt) than DSP-S. The ratio of the fragment number below 90 bp over the fragment number above 200 bp was very homogenous among both DSP-S and SSP-S profiles, being 134-fold lower with DSP-S than with SSP-S. Cir-mtDNA and cir-nDNA DSP-S and SSP-S mean size profiles of healthy individuals ranged in different intervals with periodic sub-peaks only detectable with cir-nDNA. The very low amount of cir-mtDNA fragments of short size observed suggested that most of the cir-mtDNA is poorly fragmented and appearing longer than ∼1,000 bp, the readout limit of this LP-WGS method. Data suggested that cir-nDNA is, among DNA extracted in plasma, associated with ∼8.6% of large structures (apoptotic bodies, large extracellular vesicles (EVs), cell debris…), ∼27.7% in chromatin and small EVs and ∼63.7% mainly in oligo- and mono-nucleosomes. By contrast, cir-mtDNA appeared to be preponderantly (75.7%) associated with extracellular mitochondria, either in its free form or with large EVs; to a lesser extent, it was also associated with other structures: small EVs (∼18.4%), and exosomes or protein complexes (∼5.9%).

**Conclusion:** This is the first study to directly compare the structural features of cir-nDNA and cir-mtDNA. The significant differences revealed between both are due to the DNA topological structure contained in the nucleus (chromatin) and in the mitochondria (plasmid) that determine their biological stability in blood. Although cir-nDNA and cir-mtDNA are principally associated with mono-nucleosomes and cell-free mitochondria, our study highlights the diversity of the circulating structures associated with cell-free DNA. They consequently have different pharmacokinetics as well as physiological functions. Thus, any accurate evaluation of their biological or diagnostic individual properties must relies on appropriate pre-analytics, and optimally on the isolation or enrichment of one category of their cirDNA associated structures.

## Introduction

Circulating DNA (cirDNA) possesses considerable potential for the study of both healthy subjects and patients with underlying pathological conditions (Stroun et al., 1977; Thierry et al., 2016). Thus, recent advances in the understanding of cirDNA have seen its use broadened, leading to the design of numerous specific approaches, as is testified by its numerous current clinical applications (Dennis Lo and Poon, 2003; Akirav et al., 2011; De Vlaminck et al., 2014; Fernandez-Cuesta et al., 2016; Wan et al., 2017; Zemmour et al., 2018; Pisareva et al., 2022a; Pisareva et al., 2022b). CirDNA has been intensely studied, with significant efforts being made to improve its detection and to discriminate its tissue/cells of origin, so that its diagnostic potential may be optimized (Zemmour et al., 2018; Meddeb et al., 2019a; Bronkhorst et al., 2020). CirDNA derives from both nuclear DNA (nDNA) and extrachromosomal mitochondrial DNA (mtDNA) (Murgia et al., 1992; Zhong et al., 2000; Meddeb et al., 2019a; Bronkhorst et al., 2020). However, most studies restricted themselves mainly to nuclear cirDNA (cir-nDNA), as compared to mitochondrial cirDNA (cir-mtDNA).

What is true for cir-nDNA, regarding improved knowledge of its structures leading to improved detection, should also be true for cir-mtDNA. Investigation on cirDNA structures has focused mainly on cirDNA fragmentation (fragmentomics), since elucidation of the cirDNA fragment size distribution may reveal characteristics linked to their release mechanism, as well as the protection against degradation in the blood stream, which is provided by DNA packaging in nucleoprotein/lipid complexes. While DNA is highly sensitive to DNase in a biological environment (Thierry et al., 1997; Pös et al., 2018), its highly negatively charged molecules have a significant capacity to bind, reversely condense, and pack tightly into macromolecular structures. nDNA is packed within nucleosomes and condensed in a hierarchical and tunable architecture mediated by DNA-protein interaction constituting the chromatin in Archaea and eukaryotes (Carrivain et al., 2012). Since mechanisms of extracellular DNA release may be various (NETosis, apoptosis, necrosis, active release,…), DNA-protein complexes may, in addition, be protected by blood floating particles such as apoptotic bodies, vesicles or associated with degraded cell membranes; all of them likely lead to different plasma extracted DNA pharmacokinetics (Fleischhacker and Schmidt, 2008; Thierry et al., 2016; Bronkhorst et al., 2020).

CirDNA were first considered to be mainly packed in nucleosomes (Holdenrieder et al., 2006), and several reports have shown that cirDNA associated structures have nucleosome footprints (Chandrananda et al., 2015; Ivanov et al., 2015; Snyder et al., 2016). We recently demonstrated that cirDNA are mainly compacted within mono-nucleosomes, which apparently constitute their most stable form, while di- or oligo-nucleosomes or larger pieces of cirDNA constitute a very minor fraction of its population (Sanchez et al., 2018; Sanchez et al., 2021). In addition, we demonstrated that blunted and jagged double-stranded DNA (dsDNA) of size up to 220 bp and down to 70 bp are packed in nucleosome/chromatosome particles (Sanchez et al., 2021). Quantitative PCR (Q-PCR) assisted data on cirDNA distribution corresponded to data obtained from low-pass whole genome sequencing (LP-WGS) performed using single-stranded DNA (ssDNA) library preparation (SSP) rather than dsDNA library preparation (DSP), allowing the harmonization of data obtained from both techniques (Sanchez et al., 2018).

Despite all of this, we still understand far less about the characteristics of cir-mtDNA than those of cir-nDNA, especially its topology in circulation. In a recent study performed by our team, we showed that the mitochondrial genome may be found in nearly 50,000-fold more copies than the nuclear genome in the plasma of healthy individuals (HI, (Al Amir Dache et al., 2020). This suggests the existence of stabilizing structures protecting mtDNA molecules, thus allowing the detection and quantification of cir-mtDNA in the bloodstream (Fliss et al., 2000; Kohler et al., 2009; Meddeb et al., 2019a; Otandault et al., 2020). We later demonstrated that blood contains cell-free intact mitochondria as well as cir-mtDNA (Al Amir Dache et al., 2020). Due to the lack of histone in mitochondria, there is so far no full explanation for cir-mtDNA stabilization/protection in the blood circulation.

The accurate differentiation of cirDNA of nuclear and mitochondrial origin is feasible (Kohler et al., 2009; Meddeb et al., 2019a; Safi and Najib, 2021), and may offer diagnostic information in specific physiological or pathological situations (Zong et al., 2016; Bezdan et al., 2020; Lowes et al., 2020; Otandault et al., 2020). Note, we hypothesized that the respective quantitation of cir-mtDNA and cir-nDNA may have potential cancer screening capacity (Thierry and El Messaoudi, 2015; Meddeb et al., 2019a; Tanos et al., 2020). Consequently, an elucidation of the structural features of cir-mtDNA may improve their detection and quantification. Up to now, only the presence of circulating cell-free DNA either associated with nucleosomes, or mitochondria (Al Amir Dache et al., 2020), or vesicle containing mtDNA (Li et al., 2020; Malkin and Bratman, 2020; Soltesz and Nagy, 2020) were revealed. No full characterization of cir-nDNA or cir-mtDNA topology in blood was established. This study was carried out from cir-nDNA and cir-mtDNA extracted from plasma preparation avoiding platelet activation. This warrants a proper evaluation of the topological nature and content of cir-mtDNA as demonstrated in our previous report (Roch et al., 2021) while enabling the direct quantitative comparison of their various structural features.

## Materials and methods

### Sources of blood samples

All HI signed an informed consent and their samples were supplied by the French Blood Establishment (EFS). Samples were collected using a conventional process with the use of a dedicated needle, a vacutainer tube holder and EDTA tubes. The blood samples were immediately prepared after collection. The characteristics of the seven HI from are indicated in Supplementary Table S1.

### Preanalytical work-up

Plasma is defined as a liquid isolated from blood after precluding blood clotting with an anticoagulating agent. To do so, various pre-analytical conditions might be used with a necessary removal of cells by using a centrifugation step. We herein employed different procedures previously detailed in our previous paper (Roch et al., 2021). Besides, the pre-analytical conditions for all blood samples strictly followed the guidelines we reported (Meddeb et al., 2019a). Among these, in order to measure properly cirDNA concentrations, we applied an immediate isolation of plasma following blood draw, a less than 4 h delay between blood drawing and plasma preparation, an examination of plasma aspect after the first centrifugation step, no delay before the second centrifugation step, and an assessment of blood cell DNA contamination by determining the DNA Integrity Index (DII).

#### Standard preparation protocol (SPP) for cirDNA analysis (cirDNA-SPP)

We used a standard centrifugation protocol previously validated (Chiu et al., 2001; El Messaoudi et al., 2013) to isolate plasma from EDTA tubes handled with a strict respect of pre-established guidelines (Meddeb et al., 2019a). Briefly, blood samples were consecutively centrifuged at 1,200 g (low-speed centrifugation, LS) and 16,000 g (high-speed centrifugation, HS) at 4°C for 10 min with each subsequent supernatant being used to obtain our plasma preparation. QIAamp DNA Blood Mini Kit (Qiagen) was then applied on 200 µL of this plasma preparation to extract cirDNA in a final elution volume of 80 µL, with an immediate storage of DNA extracts obtained at −20°C until use. To examine cirDNA origins, we also used a 0.22 µm Polysulfone membrane filter to set a filtration step (Al Amir Dache et al., 2020) and a −20°C freezing step between LS and HS.

#### Plasma preparation without platelet activation (PPw/oPA)

To preclude platelet activation during plasma isolation, we used a protocol previously detailed in our previous paper (Roch et al., 2021). Briefly, we collected fresh blood in dedicated tubes with a subsequent isolation of plasma through differential centrifugations: two consecutive steps at 200 g, a third centrifugation at 300 g, the subsequent addition of an anticoagulant solution containing prostaglandin E1 to the plasma, and three further centrifugations at 1,100, 2,500 and 16,000 g, all centrifugations steps being carried out for 10 min at room temperature.

#### Differential centrifugation and filtration (F) of plasma

The different plasma preparations used here are detailed in our previous paper (Roch et al., 2021). Briefly, we examined different approaches to prepare plasma from each blood sample, with variations considering centrifugation speed and/or F. After an initial 1,200 g centrifugation step, the isolated plasma was split in four equal volumes: (i), the first aliquot (LS) was not submitted to any additional treatment and considered as a control; (ii) the second one was filtered (LS+F) with a 0.22 µm filter; (iii), an additional centrifugation at 16,000 g was performed for the third one (LS+HS); and (iv), the last one was centrifuged at 16,000 g centrifugation after being stored at −20°C (LS+freezing+HS). The cirDNA was then extracted from the abovementioned plasma preparations and then analyzed by Q-PCR.

Second, the plasma was isolated at 400 g with Ficoll gradient. The supernatant was centrifuged at 16,000 g for 10 min at 4°C, (Micro Star microcentrifuge, VWR), then further centrifuged at 40,000 g for 1 h at 4°C, and finally centrifuged at 200,000 g for 2 h at 4°C (Beckman MLA-130 Ultracentrifuge Rotor). After each centrifugation step, an aliquot was performed and the cirDNA extracted from supernatants for Q-PCR analysis.

### Quantification of cir-mtDNA and cir-nDNA by Q-PCR

The Q-PCR amplification protocol used here is exhaustively detailed in our previous paper (Roch et al., 2021) and followed MIQE guidelines (Bustin et al., 2009). Every sample was submitted to a triplicate measurement of cirDNA concentrations.

Briefly, Q-PCR amplifications were performed with a 25 μL-total reaction volume. Thermal cycling started with a denaturation step, followed by 40 repeated cycles of 10 s at 90°C and 30 s at 60°C. Melting curves were obtained through a gradual increase of the temperature from 55°C to 90°C. DIFI human colorectal cancer cell line was used to calibrate the quantifications and check the efficiency of each pair of primers for which an additional negative control step was set. The primers used targeted cir-nDNA and cir-mtDNA through previously validated wild type sequences in *KRAS* gene (67 bp and 320 bp) (Mouliere et al., 2013; Mouliere et al., 2014) and in the *MT-CO3* gene (67 bp and 310 bp) (Meddeb et al., 2019a; Tanos et al., 2020). We ascertained sample concentrations from triplicate measurements through the extrapolation of our standard curves.

### Preparation of sequencing libraries

Both DSP and SSP were prepared. SSP allows the integration of ssDNA and dsDNA in the library. DSP libraries were prepared with the NEB Next^®^ Ultra™ II kit (New England Biolabs). SSP libraries were prepared with the Swift ACCEL-NGS^®^ 1S PLUS kit (Swift Biosciences). For both preparations, a minimum of 1 ng of cirDNA was engaged without fragmentation, and each kit providers’ recommendations were followed. For quality control purpose, cirDNA extracts were controlled by Bioanalyzer capillary electrophoresis and quantified by Q-PCR. For DSP, repaired A-tailed fragments were submitted to a ligation with Illumina paired-end (PE) adaptor oligonucleotides. A purification was then applied by solid-phase reversible immobilization (SPRI), followed by an enrichment by 11 PCR cycles with unique dual index primers indexing, and another SPRI purification. For SSP, to convert all DNA into single strands, a first step of heat denaturation was accomplished. Through the use of an adaptase, this protocol enables the simultaneous binding of an adapter to the end of each single-stranded fragment and the lengthening of the 3’ end of this fragment. Subsequently, a primer extension synthesized the complementary strand, followed by a SPRI purification, the ligation of a second adapter at the other end, and another step of SPRI purification. An enrichment of the product was then performed by 11 PCR cycles, followed by a final SPRI purification. An adjustment of the SPRI purification was operated to keep the small fragments around 70 bp of insert for both types of preparation. Lastly, a precise quantification by Q-PCR was applied to the libraries to be sequenced, to ensure that the appropriate DNA quantity was loaded to the Illumina sequencer, and that a minimum of 1.5 million of clusters would be obtained.

The calculation of each fragment size’s frequency, expressed in %, was carried out using the ratio of the sequenced reads to the total reads obtained in the library. The fragment size distribution length unit was base pairs (bp) when using DSP-sequencing (DSP-S), and nucleotides (nt) when using SSP-sequencing (SSP-S). To allow the comparison of data from both DSP-S and SSP-S, the size or size range were then expressed as bp (nt).

Note, these are PCR-free libraries, with no initial PCR targeted sequencing performed, given our knowledge on the previously described high cirDNA fragmentation showing an appropriate mean fragment size range for whole genome sequencing (WGS), from 150 to 160 bp (Sanchez et al., 2018).

### Size profile analysis by low-pass WGS

A NextSeq 500 Sequencing System or NovaSeq 6,000 Sequencing System (Illumina) were used to sequence all libraries as PE 100 reads. Image analysis and base calling was achieved through the use of Illumina Real-Time Analysis, using default parameters. A cut of the individual barcoded PE reads was performed with Cutadapt v1.10, removing the adapters to discard trimmed reads shorter than 20 bp. An alignment of the trimmed FASTQ files with the human reference genome (Genome Reference Consortium Human Build 38, https://www.ncbi.nlm.nih.gov/grc/human) was obtained using the Maximum Entropy Method algorithm in the Burrows-Wheeler Aligner v0.7.15. The insert sizes were then extracted from the aligned bam files with the Template Length column for all pairs of reads with an insert size between 0 and 1,000 bp.

The ratio of the number of reads at each fragment size to the total number of fragments from 30 to 1,000 bp (nt) was used to calculate the frequency of each fragment size separated by 1 bp or nt. Relying respectively on the presence of dsDNA or ssDNA, the size profiles generated by DSP-S or SSP-S were respectively expressed in bp and nt as a fragment size unit. Note, the fragment sizes are offset by 3 bp (nt) for the two methods, which is consistent with damaged or non-flush input molecules, whose true endpoints are more faithfully represented in single-stranded libraries.

DNA libraries and sequencing were performed by IntegraGen SA (Evry, France). Under the aforementioned technical conditions, the limits of detection by sequencing are estimated to be 30 bp (nt) at the lower end, and nearly 1,000 bp (nt) at the upper end. Sequencing of plasma-extracted cirDNA was performed from either DSP or SSP in plasma as previously reported (Sanchez et al., 2021).

### Statistics and drawings / statistical analysis

We used GraphPad Prism software V6.01 to fulfill statistical analysis, calculation and log transformation of relevant data. Means were compared with a Student’s t-test. Statistical significance of data was set with a two-sided p-value under 0.05. Significant p-values are indicated in the different figures by *p < 0.05, **p < 0.01; ***p < 0.001; ****p < 0.0001. Figures of cirDNA size profile as performed by LP-WGS were drawn using R studio.

## Results

We first explored cir-mtDNA’s structural features using the analytical strategy of combined Q-PCR and LP-WGS analysis, from both DSP and SSP. This successfully provided a full determination of the fractional distribution of cir-nDNA over a wide size range (Sanchez et al., 2018; Sanchez et al., 2021). Therefore, we applied this strategy to compare the cir-nDNA and cir-mtDNA size profiles.

### Low-pass WGS-based size profile of cirDNA, from plasma prepared with the cirDNA standard protocol

In HI, the mean number of reads for cir-mtDNA was 79 (range 24–207) for DSP-S and 119 (range 25–277) for SSP-S; the mean number of reads for cir-nDNA was 1,434,487 (range 1,079,717- 1,611,205) for DSP-S and 1,007,070 (range 708,192- 1,299,291) for SSP-S (Supplementary Table S2). The DSP-S and SSP-S ratios of the mean number of cir-mtDNA reads over cir-nDNA reads were 0.006% and 0.012% in HI (Supplementary Table S2). Because of the low read number of cir-mtDNA, we presented size profiles with histogram values at each fragment size up to 1,000 bp (nt).

#### Mitochondrial circulating DNA size profile

**FIGURE 1:**
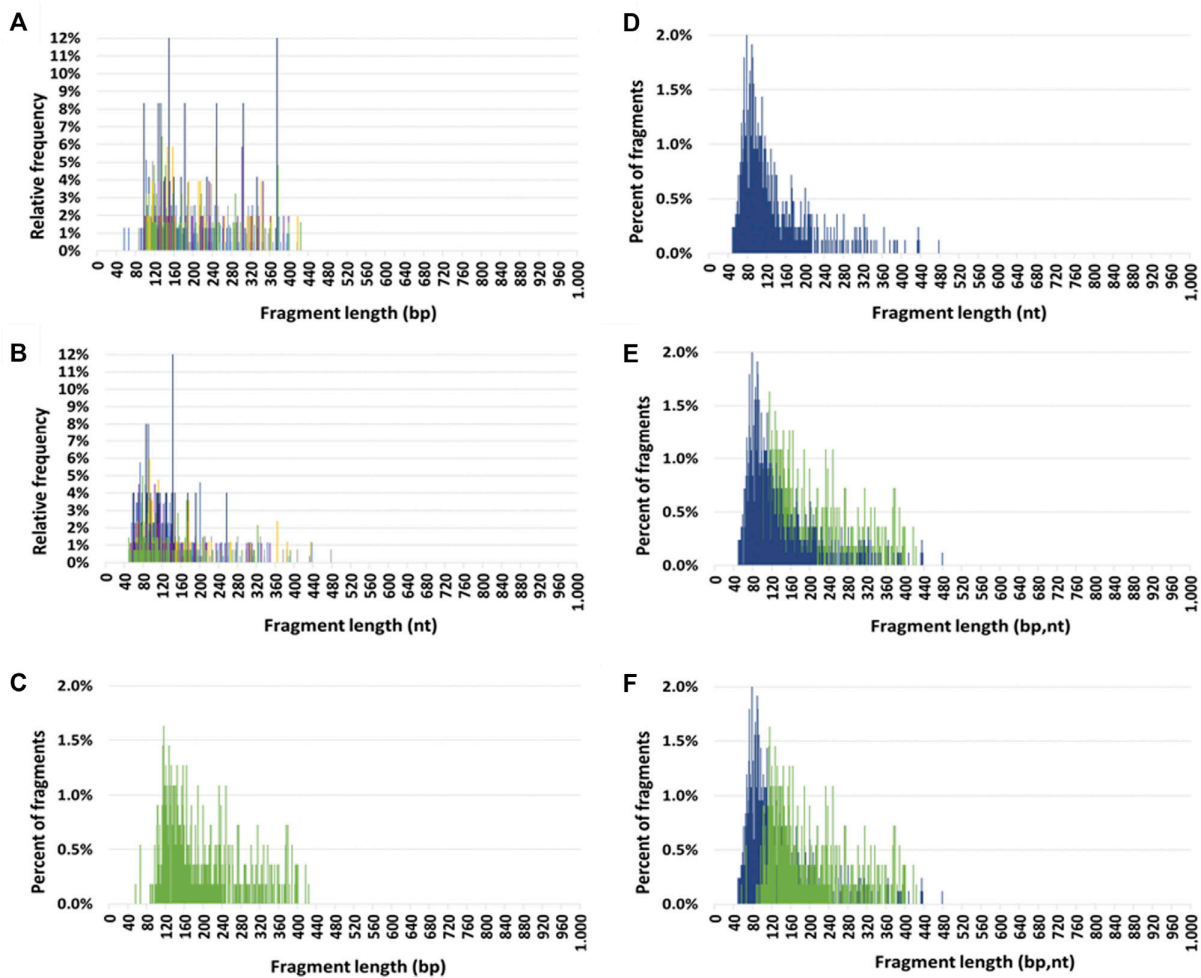
Circulating mitochondrial DNA (cir-mtDNA) size profile in healthy individuals. Relative frequencies of cir-mtDNA fragments from seven subjects (seven colours) as determined by low-pass WGS: **(A)**, DSP sequencing (DSP-S); **(B)**, SSP sequencing (SSP-S). Mean percent of fragments of cir-mtDNA from the seven subjects as determined by DSP-S [green, **(C,E,F)**] and SSP-S [blue, **(D,E,F)**]: DSP-S alone **(C)**, SSP-S alone **(D)**, SSP-S in foreground and DSP-S in background **(E)**, DSP-S in foreground and SSP-S in background **(F)**.

The seven HI size profiles obtained by DSP-S and SSP-S are pasted over each other in Figures 1A, B, respectively. Using DSP-S, relative frequencies distributed from 55 to 425 bp (Figure 1A), with a sharp increase up to 120 bp, a subsequent progressive decrease with no fragments above 425 bp, and a slightly higher number of fragments from 90 to 260 bp. Using SSP-S, relative frequencies distributed from 50 to 475 nt (Figure 1B), with a sharp increase up to 80 nt, followed by a progressive decrease in the number of fragments. Two distinct populations appeared in the profiles obtained by SSP-S: one monomodal population from 50 to 150 nt, and a population smear from 150 to 475 nt, with no fragments above 475 nt (Figure 1B). Cir-mtDNA size profiles appeared to be homogeneous among the seven HI, both for DSP-S and SSP-S (Figures 1A, B). The majority of detected fragments in HI DSP-S mean size profiles distributed from 90 to 260 bp (77.9% of total fragments), with the number of fragments sharply increasing from 90 to 120 bp, peaking at 120 bp, and decreasing slowly in the range beyond that (Figure 1C). The majority of detected fragments in HI SSP-S mean size profiles distributed from 50 to 150 nt (74.4% of total fragments), with the number of fragments sharply increasing from 50 to 80 nt, peaking at 80 nt, sharply decreasing from 80 to 150 nt, then decreasing slowly beyond that (Figure 1D). Differences emerged when the DSP-S and SSP-S mean size profiles were compared (Figures 1E, F). First, DSP-S mean size profiles distributed on a slightly shorter range (90–425 bp) than SSP-S (50–475 nt). Maximal values as determined by both methods ranged from about 100 to 150 bp for DSP-S and from 70 to 100 nt for SSP-S. Thus, SSP-S detected a higher number of low-sized sequences with a high mean frequency of fragments between 50 and 120 nt (61.0% of total fragments), while DSP-S showed a low number of fragments in this range (16.7% of total fragments).

#### Comparison of mitochondrial and nuclear circulating DNA size profiles

**FIGURE 2:**
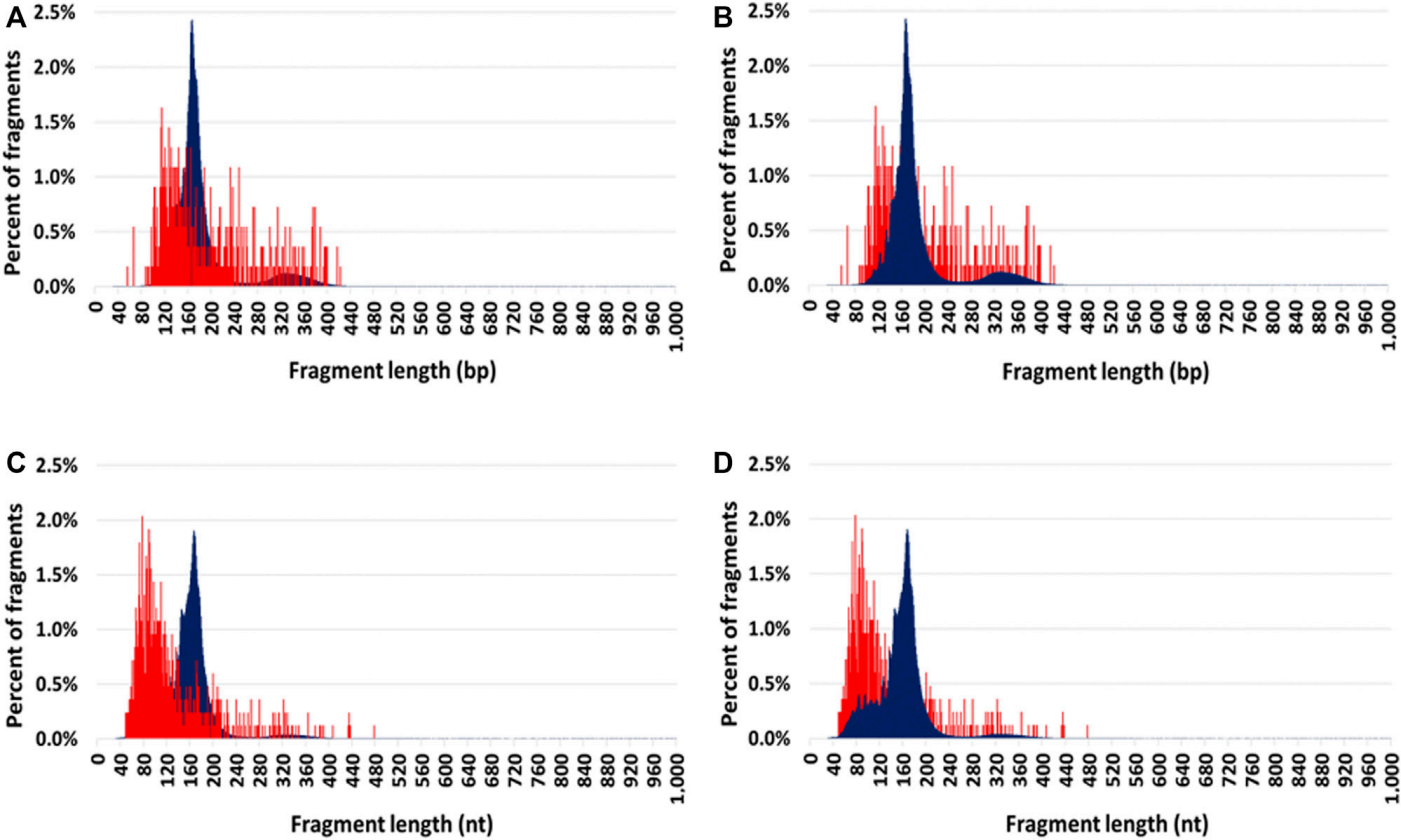
Comparison of the mean size profiles of circulating mitochondrial DNA (cir-mtDNA) and circulating nuclear DNA (cir-nDNA) from seven healthy individuals with alternate foreground. Low-pass WGS mean size profiles of cir-mtDNA (red) and cir-nDNA (dark blue) obtained with DSP-sequencing **(A,B)** and SSP-sequencing **(C,D)**: cir-mtDNA in foreground and cir-nDNA in background **(A,C)**; cir-mtDNA in background and cir-nDNA in foreground **(B,D)**.

Cir-mtDNA and cir-nDNA DSP-S mean size profiles of HI ranged from 55 to 425 bp and 85 to 420 bp, respectively (Figures 2A, B). The DSP-S cir-mtDNA profile of HI exhibited a major monomodal population, mostly ranging between 90 and 260 bp, peaking at 120 bp, and a population appearing as a smear above 260 bp (21.0% of total fragments). The DSP-S cir-nDNA profile of HI had a major population between 85 and 260 bp (89.2% of total fragments), peaking at 166 bp (2.5% of total fragments); a minor population was also detectable between 261 and 420 bp (10.5% of total fragments), with no fragment detected above 420 bp. Periodic sub-peaks every 10 bp from 102 to 152 bp were detectable with cir-nDNA in contrast to cir-mtDNA. Cir-mtDNA and cir-nDNA SSP-S fragments size profile of HI ranged respectively from 50 to 475 nt and 40 to 400 nt (Figures 2C, D). The cir-mtDNA SSP-S profile of HI had a major monomodal population between 50 and 150 nt, peaking at 80 nt, while there was a slowly decreasing population smear from 151 to 475 nt (25.2% of total fragments). Cir-nDNA SSP-S mean size profile had a major population between 45 and 260 nt (96.3% of total fragments), peaking at 166 nt, corresponding to nearly 2.0% of total fragments. The number of fragments plateaued between 70 and 120 bp at nearly 0.4% of total fragments. A very small population was observed between 261 and 400 nt (3.4% of total fragments). Periodic sub-peaks every 10 nt from 53 to 144 nt only existed with cir-nDNA.

We observed a nearly 40-fold lower frequency of cir-mtDNA fragments <90 bp with DSP-S (mean+/-SD = 0.9+/-1.2%) than with SSP-S (mean+/-SD = 33.5+/-4.9%) (Supplementary Table S3A and Supplementary Table S3B). Additionally, the frequencies of fragments above 200 bp (nt) were homogenous among DSP-S (Supplementary Table S3A) and SSP-S cir-mtDNA size profiles (Supplementary Table S3B). However, the frequency of fragments above 200 bp (nt) was 3-fold higher with DSP-S (mean+/-SD = 38.1+/-4.2%) compared with SSP-S (mean+/-SD = 12.3+/-4.0%), meaning a higher number of fragments above 200 bp (nt) were obtained with DSP-S, compared with SSP-S. The ratio of the frequency below 90 bp over the frequency above 200 bp (Frequency <90)/(Frequency >200) was extremely homogenous among both DSP-S and SSP-S profiles. Nevertheless, this ratio was 134-fold lower with DSP-S (mean+/-SD = 0.02+/-0.03) than with SSP-S (mean+/-SD = 3.3+/-2.1).

We observed a very low amount of cir-mtDNA fragments of short size suggesting that most of the cir-mtDNA is poorly fragmented and appearing longer than ∼1,000 bp, the readout limit of this LP-WGS method. Since we found that conventional plasma preparation results in *in vitro* platelet activation (Roch et al., 2021) and consequently to mitochondria extracellular release (Meddeb et al., 2019a; Al Amir Dache et al., 2020), we used a plasma procedure precluding platelet activation. This enabled us to quantitatively compare the forms and structures of plasma extracted DNA floating in the blood circulation. In order to estimate the cirDNA various structural forms, plasma fractions were subjected to physical examination.

### Physical examination of plasma prepared by various methods

**FIGURE 3:**
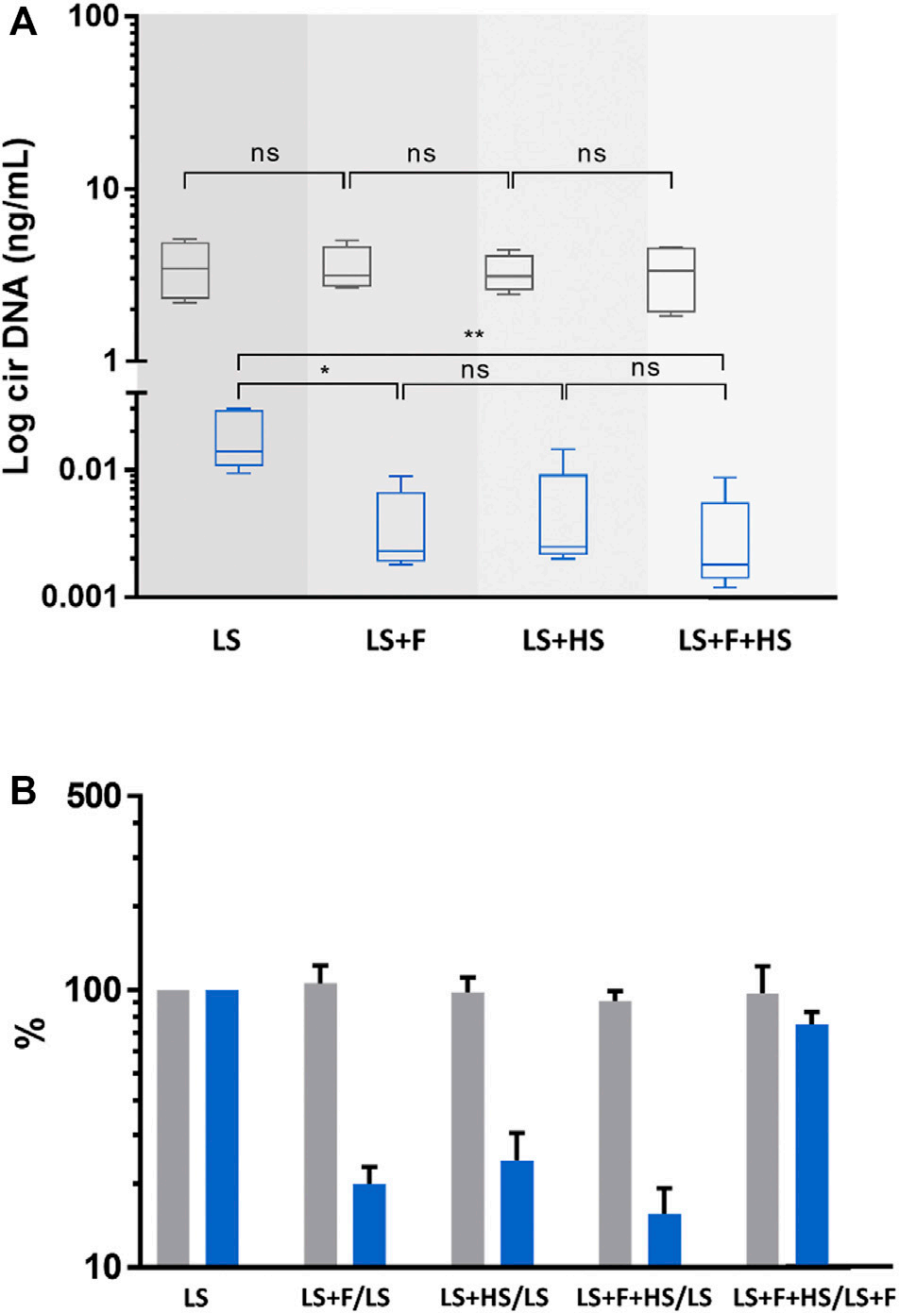
Comparison of the concentrations and variations of circulating nuclear (cir-nDNA, grey) and mitochondrial DNA (cir-mtDNA, blue) from 5 healthy individuals, depending on the physical process applied. The preparation protocol used was without platelet activation (PPw/oPA, **(A,B)**. The physical procedures applied were low-speed centrifugation (LS), filtration (F), and high-speed centrifugation (HS). Samples were either treated with LS only or with LS combined either with F (LS+F) or with HS (LS+HS). The respective variation of the cirDNA concentration in each group was obtained comparing each combination with LS only, used as a reference. Results are presented as boxplots **(A)** and histograms **(B)**. ns, non-statistically significant; *p < 0.05, **p < 0.01; ***p < 0.001.

**FIGURE 4:**
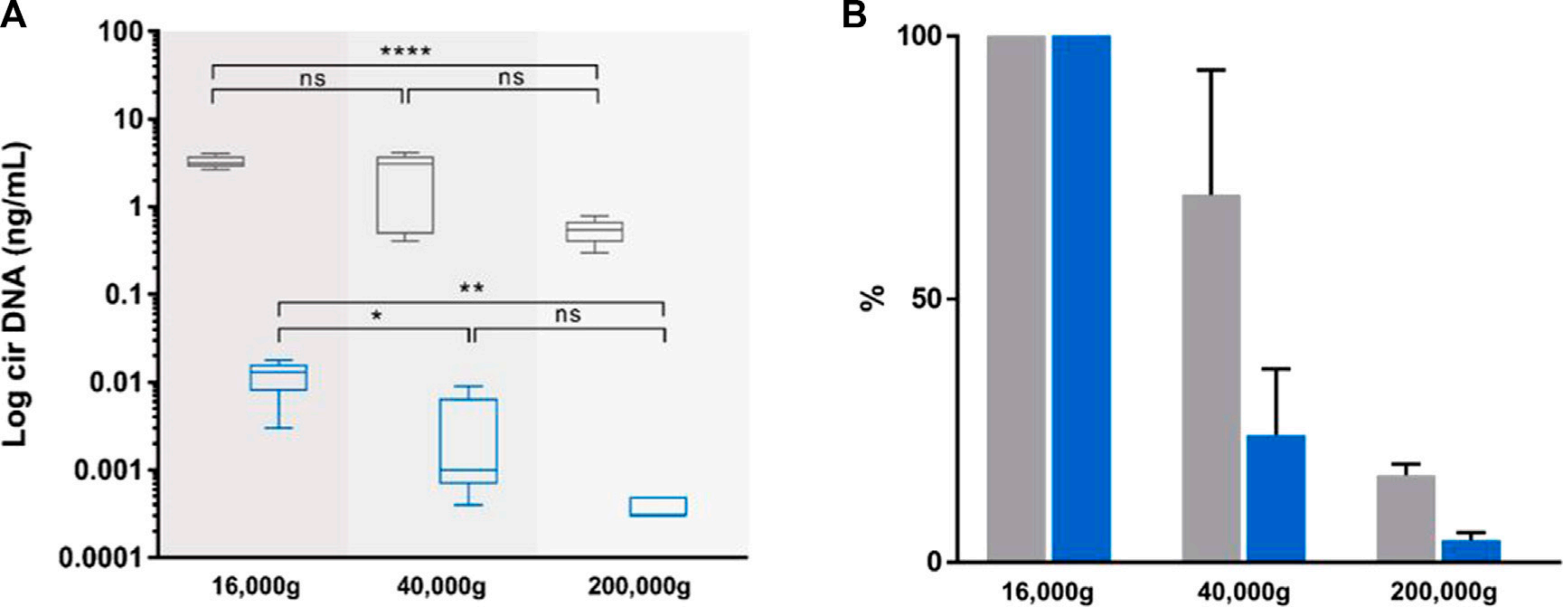
Effect of incremental centrifugation speed on plasma from five healthy individuals. Comparison of the concentrations [**(A)**, ng/mL], and variations [**(B)**, %)] of circulating nuclear DNA (cir-nDNA, grey) and circulating mitochondrial DNA (cir-mtDNA, blue) in plasma as prepared by the protocol without platelet activation. cirDNA concentration was determined in the supernatant following the subsequent centrifugation steps of the respective supernatant at 16,000 g, 40,000 g and 200,000 g. ns, non-statistically significant; *p < 0.05, **p < 0.01; ****p < 0.0001.

Cir-nDNA and cir-mtDNA extracts from both preparation protocol, respectively cirDNA-SPP and PPw/oPA were partitioned to elucidate the fractions of the various cirDNA structural forms. During this plasma preparation process, a complementary step with LS alone or with a subsequent F was also added to potentially evaluate the presence of particles. It should be noted that two of the seven subjects had not enough plasma to perform the abovementioned experiments, described in Figure 3 and Figure 4.

#### Effect of the use of HS, F and freezing step on plasma preparation (cirDNA-SPP)

As described in our previous paper (Roch et al., 2021), cir-mtDNA supernatant concentrations were significantly modified by F and HS, applied after LS (LS+F or LS+HS), and freezing, performed between LS and HS (LS+freezing+HS). Both LS+F and LS+HS led to a significant decrease, compared to supernatant from LS (Supplementary Table S4A), while freezing resulted in a significant increase (12-fold) of the cir-mtDNA amount in LS+F+HS supernatant, compared with LS+HS.

Supernatant derived cir-nDNA mean concentrations following LS, LS+F, LS+HS and LS+freezing+HS were 5.28, 4.53, 7.25 and 9.27 ng/mL, respectively (Supplementary Table S4A). No significant effect was observed on cir-nDNA level due to either F or HS. Adding a freezing step between LS and HS increased by 28% the cir-nDNA concentration, which was not statistically significant. The proportions of cir-mtDNA mean concentrations among the total cirDNA mean concentrations were 19.5%, 0.3%, 0.1% and 0.9% following LS, LS+F, LS+HS and LS+freezing+HS, respectively (Supplementary Table S4A). All concentration values are described in Supplementary Table S4A.

#### Effect of the use of HS and F on plasma preparation (PPw/oPA)

Cir-mtDNA concentrations following LS, LS+F, LS+HS and LS+HS+F were 0.019, 0.004, 0.005, and 0.003 ng/mL, respectively. The obtained values after F and HS of the plasma following LS showed an 80% (*p* = 0.012) and 75.7% decrease (*p* = 0.010) of cir-mtDNA levels (Figures 3A, B). By contrast, cir-nDNA level was not significantly altered by F or HS (Figures 3A, B) with concentrations following LS, LS+F, LS+HS and LS+HS+F of 3.55, 3.51, 3.28 and 3.28 ng/mL, respectively. Following LS, LS+F, LS+HS and LS+F+HS, the fractions of cir-mtDNA copies among the total cirDNA copies were 0.5, 0.1, 0.2 and 0.1%, respectively. All concentration values are described in Supplementary Table S4B.

#### Estimation of extracellular vesicles (EVs) associated cir-mtDNA proportion

While the plasma preparation without platelet activation provides highly qualitative information, its stringency, such as the numerous centrifugation steps, precludes accurate quantitative assessment of the cir-mtDNA. As a first step, we therefore used a Ficoll plasma preparation, and performed subsequent 16,000, 40,000 and 200,000 g centrifugations of the respective supernatant, which found about 3.7 × 10^6^, 9.2 × 10^5^ and 1.1 × 10^5^ copy number of mtDNA per mL, respectively. As for cir-nDNA, we found 1,001, 705 and 165 copy number of nDNA per mL, respectively. Considering cir-mtDNA, a significant statistical difference was only observed between the groups of plasma following the 16,000 g and the 40,000 g and between those following the 16,000 g and the 200,000 g centrifugations (*p* = 0.04, Figure 4), while such a difference only appeared between those following the 16,000 g and the 200,000 g centrifugations for cir-nDNA (*p* < 0.0001, Figure 4). From the total amount of mtDNA previously present in the 16,000 g supernatant, 24.3% and 4.2% of cir-mtDNA remained in the 40,000 g and 200,000 g supernatant, whereas 63.7% and 10.6% of cir-nDNA remained in the 40,000 g and 200,000 g supernatant (Supplementary Table S4C). The proportions of cir-mtDNA copies among the total cirDNA copies were 0.4, 0.1 and 0.1% following the 16,000 g, 40,000 g and 200,000 g centrifugation, respectively. All concentration values are described in Supplementary Table S4C.

## Discussion

Beyond quantitative observations (Meddeb et al., 2019a; Otandault et al., 2020; Roch et al., 2021), cir-nDNA analysis has benefited greatly from the understanding of its structural features acquired from their fragment size profile or fragmentomics. Works from Ellinger et al. (Ellinger et al., 2008) and Diehl et al. (Diehl et al., 2005) revealed that fragments may be of a size lower than 180 bp; that was previously thought to be the lowest size (Jahr et al., 2001), as it corresponds to that existing in a mononucleosome plus linker. Subsequently, we were the first to show that cirDNA is mostly highly fragmented, with a significant part of those fragments being of sizes down to 60 bp (Mouliere et al., 2013). Subsequent works using WGS analysis confirmed this in greater detail (Fan et al., 2010; Chandrananda et al., 2015; Ivanov et al., 2015; Jiang et al., 2015; Snyder et al., 2016). In addition, WGS-assisted fragmentomics revealed nucleosome footprints, which indicated that detected short fragments (30–400 bp) derive from the nucleosome around which cirDNA is packed and relatively stabilized in the blood compartment (Ivanov et al., 2015; Snyder et al., 2016; Sanchez et al., 2021). Furthermore, Shendure’s team (Snyder et al., 2016) inferred tissue of origins from nucleosome occupancy as determined by WGS. We also described how cirDNA distribution in cancer patient plasma is mostly represented (67–80%) by mono-nucleosome associated cir-nDNA (Sanchez et al., 2021). In addition, Q-PCR assisted data on cirDNA distribution corresponded to WGS performed using SSP-S rather than DSP-S. This allowed the harmonization of data obtained from Q-PCR and WGS, since both SSP-S and Q-PCR use ssDNA as a template (Sanchez et al., 2018; Sanchez et al., 2021). A procedure combining LP-WGS and Q-PCR points to the possibility of identifying all kinds of nicks on fragments under 1,000 bp, and may also provide clues about potential cirDNA stabilizing structures in blood. Moreover, by adding a Q-PCR approach, we have given ourselves the possibility of learning more about fragments over 1,000 bp, and thus extending our comprehension of the size profile and the level of fragmentation of cir-nDNA (Sanchez et al., 2018; Sanchez et al., 2021). As previously demonstrated (Sanchez et al., 2021), LP-WGS allows the inference of the presence of blunt or jagged dsDNA by comparing DSP-S and SSP-S. This process has also provided the following observations: (i), jagged dsDNA appeared by far the most present cirDNA structural forms; (ii), the lowest cirDNA fragment size is about 70 bp; and (iii), this might be due to the low hybridization forces of the lower-sized jagged fragments leading to their peeling off from histone/nucleosome particles (Sanchez et al., 2021). Altogether, our data suggest that the proportion of cir-nDNA inserted in mono-nucleosomes, di-nucleosomes, and chromatin of higher molecular size (>1,000 bp) in HI ranges from 67% to 80%, 9% to 12%, and 8% to 21%, respectively (Sanchez et al., 2021).

Considering how useful this combined analytical approach had proved, we then applied it in an investigation of cir-mtDNA structural features. To this end, we used the cirDNA-SPP and a stringent clinically validated DNA extraction process, and followed our previously published guidelines (Meddeb et al., 2019a). As we observed with cir-nDNA from LP-WGS, cir-mtDNA size profiles appeared homogenous and reproducible in the plasma of HI, whether detected by DSP-S or SSP-S (Figure 1); and in both cases, an overwhelming majority of fragments were less than 400 bp in size. This suggests that this population of short fragments is the result either of degradation by-products or of some means of cir-mtDNA protection. However, we found periodic distribution neither with DSP-S nor with SSP-S, while the DSP-S and the SSP-S mean size profiles differed significantly (Figure 1). With SSP-S, we detected a higher number of short fragments in the 50–120 nt interval, an earlier peak at 80 nt, and a faster decrease of mean percent of fragments after 80 nt, as compared with DSP-S. DSP-S showed a higher proportion of fragments in the 120–200 bp interval, with a later and lower peak, around 120 bp, and a subsequent slower decrease of mean percent of fragments after 120 bp. The significant differences between DSP-S and SSP-S may reveal a structural difference, since observation of a short population implies the protection of DNA strands by a stabilizing structure, as observed in cir-nDNA associated with nucleosomes. The clear differences existing between DSP-S and SSP-S profiles led us to believe that shorter ssDNA fragments appeared following SSP-S from jagged dsDNA, with at least one nick in one DNA strand still bound to the structure protecting the DNA from nuclease attack.

Only a few existing reports contributed to characterize cir-mtDNA structure. Chiu et al. (Chandrananda et al., 2015) initiated efforts towards this goal, and indirectly showed that cir-mtDNA may consist of both particle-associated and non-particle-associated forms of mtDNA in plasma. By PE sequencing analysis, a report (Chandrananda et al., 2015) indicated that microbial and mtDNA are exposed to similar degradation processes, having observed that the fragmentation profiles of microbial and mtDNA in plasma were highly similar. Like Jiang et al. (Jiang et al., 2015), they also reported that cir-mtDNA size distribution is consistently shorter than cir-nDNA. Since mtDNA cannot be packed as nDNA with histones within nucleosome, both stated that cir-mtDNA is more susceptible to enzymatic degradation, given that its size profile reflects traces of wide range-sized mtDNA under dynamic degradation. Furthermore, Chandrananda et al. found that SSP-S is more effective than DSP-S at recovering bacterial, viral and mitochondrial cirDNA (Chandrananda et al., 2015). These results may challenge the common paradigm, which considers fragments of cir-mtDNA below 400 bp as consisting only of degradation products (Chandrananda et al., 2015). Burhnam et al. later confirmed this statement (Burnham et al., 2016).

Consequently, our findings lead to a profound revision of previous assumptions regarding the significance of the cir-mtDNA amounts detected in our and other previous works (Chiu et al., 2003; Jiang et al., 2015; Meddeb et al., 2019a; Bezdan et al., 2020; Grabuschnig et al., 2020; Li et al., 2020; Liu et al., 2020; Hägg et al., 2021). First, it can no longer be considered valid to examine short cir-mtDNA fragments (in particular under 1,000 bp) using DNA fragmentation index as determined by Q-PCR (Ellinger et al., 2009; Zonta et al., 2015; Meddeb et al., 2019b; Tanos et al., 2020) or using WGS (Chandrananda et al., 2015; Jiang et al., 2015)) when considering the overall cir-mtDNA population, since it accounts for a very tiny part of the cir-mtDNA amount. Despite their low plasma content, mtDNA blood degradation products might nonetheless be relevant when considering differential enzymatic activity which may occur in certain physiological or pathological conditions, as suggested for cir-nDNA (Serpas et al., 2019; Sanchez et al., 2021).

Uncertainty as to the nature of the short cir-mtDNA fragments population nonetheless appears of minor importance, since its proportion to the total cirDNA fragments is very low: (i), the cir-mtDNA mean number corresponded to 0.006% and 0.012% of the total cirDNA fragments (Supplementary Table S2), when using DSP-S and SSP-S, respectively; (ii), there are nearly 50,000-fold more copies of the mitochondrial genome in plasma (Al Amir Dache et al., 2020), which accounts for 10–25% of the total cirDNA mass content; and (iii), the DII as determined by Q-PCR showed that nearly all cir-mtDNA is over 310 bp (Roch et al., 2021). Inside cells, mtDNA is bound with mitochondrial transcription factor A (TFAM), constituting nucleoprotein complexes called nucleoids. As previously published (Bogenhagen, 2012), the copy number of mtDNA contained in a single nucleoid may vary between 1.4 and 7.5, depending on the cell type and the method used. TFAM are protein factors that may theoretically also protect cir-mtDNA in blood circulation. However, these types of nucleoprotein complexes are sedimenting following 100,000/200,000 g centrifugation speed, and therefore could represent at the most 4.4% of total cir-mtDNA, as shown here. These complexes are thus to be considered as a very minor part of cir-mtDNA and could not significantly influence our results.

In contrast, cir-nDNA is highly fragmented with a DII showing a proportion of the number of detected fragments above 320 bp ranging from 10 to 20% (Meddeb et al., 2019a; Sanchez et al., 2021). Since practically no cir-mtDNA fragments were detected between 310 bp and 1,000 bp (Figure 1), which is the practical upper limit of read-out in LP-WGS sizing using either DSP-S or SSP-S, we infer that most cir-mtDNA fragments are over 1,000 bp. Our data converge with that of several other studies. For instance, using a Q-PCR-based assay, Ellinger et al. reported a plasma mtDNA integrity between 0.5 and 1.0 (Ellinger et al., 2009). Data obtained in this study confirmed our previous observation that the cir-mtDNA DII is always close to 1; this indicates that cir-mtDNA is not or very poorly fragmented (Thierry and El Messaoudi, 2015). Note, we previously observed a much higher stability of cir-mtDNA compared to cir-nDNA extracted from serum-containing media of cultured cells (Otandault et al., 2020). Our postulate is that cir-mtDNA is either unfragmented when included in mitochondria (free or encapsulated in EVs) or very fragmented as observed in our WGS-based data.

The three main methods available for determining DNA fragment length are LP-WGS, Q-PCR, and Agilent capillary electrophoresis; however, these are limited in respect to (i) the practical upper limit of read-out around 1,000 bp (LP-WGS); (ii) the general low accuracy and precision (capillary electrophoresis); and (iii), the capacity to compare size fragments over 1,000 bp (Q-PCR). In order to decipher the major structural forms of the detected cir-mtDNA, we carried out a variety of experiments, based on physical examination and various plasma preparations. This led to a number of striking observations. First, we observed that, compared to the consecutive two-step centrifugation, the frozen storage step between LS and HS, as conventionally performed in the cirDNA-SPP, led to an increase by at least 10-fold of cir-mtDNA plasma concentration, whereas similar levels of cir-nDNA concentration were found (Supplementary Table S4A). It should be noted that very recent work has reported similar observations (Wong et al., 2021). This means that freezing disrupts some structures in a way that leads to the release of cir-mtDNA but not cir-nDNA. Second, HS dramatically reduced the resulting plasma cir-mtDNA concentration by about 99%, as compared to the 1,200 g plasma supernatant, whereas both supernatants revealed equivalent levels of cir-nDNA (Figures 3A, B, Supplementary Table S4A). This means that cir-mtDNA are mostly contained in or associated with structures whose densities correspond to that of cell organelles, membrane debris or apoptotic bodies. Third, filtration of the plasma supernatant obtained following a 1,200 g centrifugation resulted in a loss of nearly 99% of detected cir-mtDNA, whereas no significant change was observed for cir-nDNA (Figures 3A, B, Supplementary Table S4A). This means that most of the cir-mtDNA are associated with structures whose size is over 0.22 µm. Note, our observations point to the existence of particles containing mtDNA in the circulation, as it has been previously indicated by plasma filtrates (Chiu et al., 2003; Malkin et al., 2022). In addition, our data agree with Arance-Criado’s observation (Arance-Criado et al., 2020), in HI, of a 99.4% decrease of cir-mtDNA following the HS step using a protocol equivalent to the cirDNA-SPP (Supplementary Table S4A). Taken together, these data suggest that nearly all detected cir-mtDNA derive from larger or denser biological structures, in contrast to cir-nDNA.

Aside from circulating cell-free mitochondria (cir-exMT) origin (Al Amir Dache et al., 2020), plasma-derived mtDNA could originate from other structures: remaining cells, in association with cell debris or membranes, mitochondria, or EVs (Malkin et al., 2022). While exosomes, and apoptotic bodies could be considered as EVs, we arbitrarily differentiated here four types of vesicles: large EVs, small EVs, exosomes, and apoptotic bodies. They may be differentiated based upon their biogenesis, release pathways, content, and function (Doyle and Wang, 2019), and may show significant differences in size. Microvesicles, exosomes and apoptotic bodies typically range from 100 nm up to 1 μm, <200 nm and >1 µm in diameter, respectively (Doyle and Wang, 2019). Differential centrifugations allow their isolation: apoptotic bodies at a g-force of approximately 2,000 g; microvesicles at 10,000–30,000 g; and exosomes by ultracentrifugation at 100,000–200,000 g. We may assume that EVs could contain either extracellular mitochondria (exMT) or fragmented mtDNA genome.

Using sequencing analysis, a previous report demonstrated the presence of only intact full mitochondrial genomes in the plasma cirDNA fraction (Newell et al., 2018), confirming our previous observation (Al Amir Dache et al., 2020). They speculate that mtDNA could be protected from degradation by circulating nucleases due to either EVs encapsulation or the circular nature of mtDNA potentially delaying its degradation (Newell et al., 2018). This study does not account for mtDNA platelet origin, however, as they used an SPP equivalent process. Based upon our observation that the proportion of cir-mtDNA of size below 1,000 bp is very weak (Roch et al., 2021), we infer that fragmented mtDNA between 1,000 bp and 16,000 bp, the approximate mitochondria full length genome, is barely associated with EVs (<0.5%). In contrast, our data based on the PPw/oPA revealed that 1.7% of the cir-mtDNA is associated with exosomes, and 18.4% mainly associated with small EVs. Thus, when taking together fragmentomics and plasma fractionation data, we infer that a fraction, at least, of EVs contains mitochondrial full length circular DNA or mitochondria particles that could be internally or externally associated with EVs. Thus, we do not preclude the possibility that mitochondria particle free mtDNA may exist in blood circulation in association with EVs, as reported previously (Malkin et al., 2022). However, its amount corresponds to a minor fraction of the total cir-mtDNA. In previous work (Al Amir Dache et al., 2020), our transmission electronic microscopy examinations showed no evidence of exMT encapsulated in or associated with bilayer phospholipidic vesicles or membranes. Moreover, our study combining LP-WGS and Q-PCR analysis showed that fragmented mtDNA of size below 1,000 bp exist in extremely small quantities (<1%, (Roch et al., 2021)). Consequently, our data associated with others (Chiu et al., 2003; Newell et al., 2018; Al Amir Dache et al., 2020) show that mtDNA detected in plasma correspond quasi-exclusively to cir-exMT.

By combining specific DNA quantification of cirDNA extracts deriving from plasma preparations avoiding platelet activation submitted to differential centrifugation and/or physical examination, our work reveals profound differences between cir-mtDNA and cir-nDNA in terms of size distribution, structure and mechanism of release. Thus, our data suggest that cir-nDNA is associated with ∼8.6% of large structures (apoptotic bodies, large EVs, cell debris…), ∼27.7% in chromatin and small EVs and ∼63.7% in exosomes, protein complexes and oligo- or mono-nucleosomes (Table 1). This was concordant with a previous report (Neuberger et al., 2021). In contrast, cir-mtDNA appeared preponderantly associated with extracellular mitochondria either free or in large EVs fraction (∼75.7%), while being associated with small EVs (∼18.4%), and with exosomes or protein complexes (∼5.9%) (Table 1).

**TABLE 1:** Suggested repartition of structures containing circulating DNA (cirDNA) in plasma. cirDNA content was measured in supernatant after successive centrifugations at different speeds, respectively 400 g with Ficoll gradient (initial copy number); 16,000; 40,000 and 200,000 g following the preparation protocol without platelet activation (PPw/oPA). cirDNA content in the pellet was inferred from this of the supernatant at each step. While exosomes, and apoptotic bodies could be considered as extracellular vesicles (EVs), we arbitrarily differentiate here four types of vesicles: large EVs, small EVs, exosomes, and apoptotic bodies. A: nuclear cirDNA (cir-nDNA); B: mitochondrial cirDNA (cir-mtDNA); mtDNA, mitochondrial DNA; nDNA, nuclear DNA; EVs, extracellular vesicles; oligoNsomes, oligonucleosomes.

A			nDNA bearing structures		From PPw/oPA
	Initial copy number (copy/mL plasma)				1,100
	Initial concentration (ng/mL plasma)				3.55
Differential centrifugation	16,000 g	Pellet	Dead cells	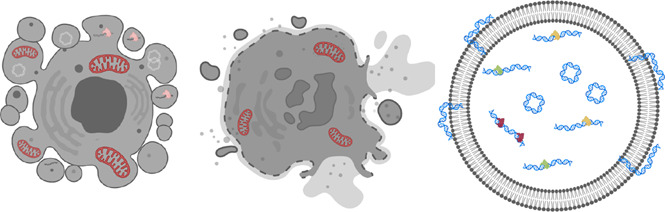	∼ 8.6%
			Associated to cell debris		
			Associated to membranes		
			Apoptotic bodies		
			Large EVs		
		Supernatant	Small EVs	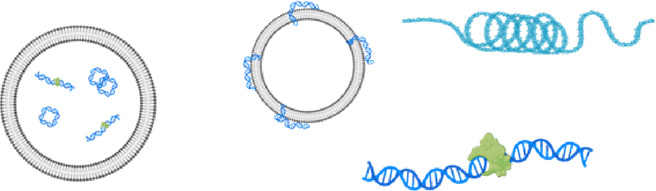	∼ 91.4%
			Exosomes		
			Protein complexes		
			Chromatin		
	40,000 g	Pellet	Small EVs	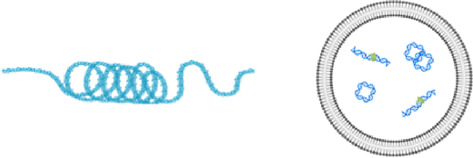	∼ 27.7%
			Chromatin		
		Supernatant	Exosomes	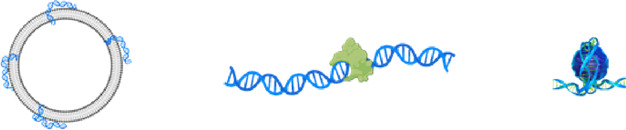	∼ 63.7%
			Protein complexes		
			Mono/OligoNsomes		
	200,000 g	Pellet	Exosomes	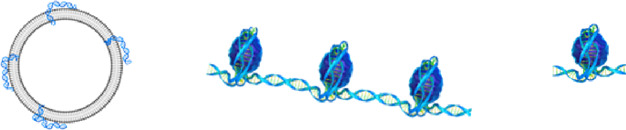	∼ 53.1%
			Mono/OligoNsomes		
		Supernatant	Protein complexes	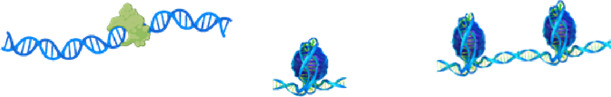	∼ 10.6%
			Mono/OligoNsomes		

B			mtDNA bearing structures		From PPw/oPA
	Initial copy number (copy/mL plasma)				5.6 millions
	Initial concentration (ng/mL plasma)				0.019
Differential centrifugation	16,000 g	Pellet	Dead cells	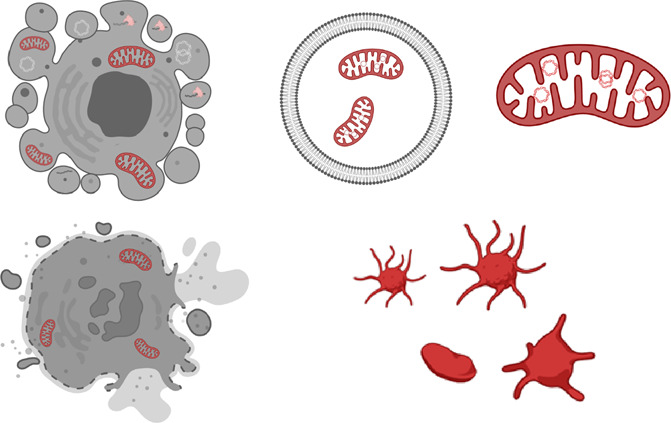	∼75.7%
			Associated to cell debris		
			Associated to membranes		
			Mitochondria		
			Apoptotic bodies		
			Large EVs		
			Platelets		
		Supernatant	Small EVs	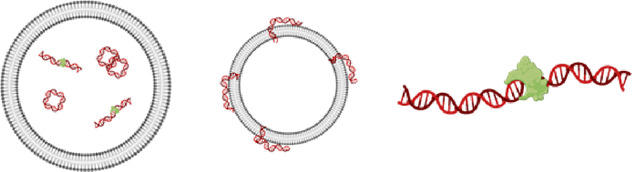	∼ 24.3%
			Exosomes		
			Protein complexes		
	40,000 g	Pellet	Small EVs	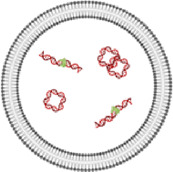	∼ 18.4%
		Supernatant	Exosomes	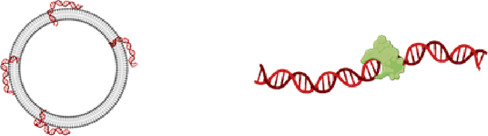	∼ 5.9%
			Protein complexes		
	200,000 g	Pellet	Exosomes	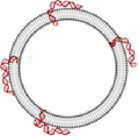	∼ 1.7%
		Supernatant	Protein complexes	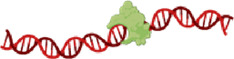	∼ 4.2%

While the plasma prepared without platelet activation contained much less cir-mtDNA (67-fold less), exMT nonetheless still represents the preponderant fraction of the total detected cir-mtDNA amount, compared to mtDNA containing EVs (Table 1). The paucity of the cir-mtDNA encapsulating microparticles/exosomes (Table 1) clearly confirms the need for specific isolation methods for their examination.

We believe that certain controversies in previous literature arose from improper conclusions based on a confusion between cir-mtDNA and cir-nDNA, and the belief that at least part of their release derive from the same mechanisms. Consequently, we propose that future studies should systematically include a measurement of both entities, to circumscribe their respective physiological impact and diagnostic power. By comparison with cir-nDNA, the structural features of cir-mtDNA appear more complex and diverse. This is principally due to the release of exMT in interstitial milieu or blood circulation from various cell types, and due to the lack of stabilizing mitochondrial components that would enable protection from extracellular nuclease degradation. Our work suggests the preponderance of cir-nDNA in mononucleosomes and of cir-mtDNA in exMT, highlighting their profound differences as regards their circulating structural forms. While the pre-analytics currently used for cir-nDNA appear satisfactory (Meddeb et al., 2019a), our data highlight the need for specific pre-analytics for cir-mtDNA. In addition, cir-nDNA and especially cir-mtDNA are both associated with different structures that might have significant differences in their respective diagnostic potentials. It is therefore necessary to better extend our knowledge of cirDNA structures of origin, and to standardize the preparation of biological material, in order to fully determine and optimize the promise of cirDNA in clinical and routine settings.

## Data Availability

The raw data supporting the conclusions of this article will be made available by the authors, without undue reservation.

## Abbreviations

cirDNA, circulating DNA; cirDNA-SPP, cirDNA standard preparation protocol; cir-exMT, circulating cell-free mitochondria; cir-mtDNA, circulating mitochondrial DNA; cir-nDNA, circulating nuclear DNA; DII, DNA integrity Index; dsDNA, double-stranded DNA; DSP, dsDNA library preparation; DSP-S, DSP-sequencing; EVs, extracellular vesicles; exMT, extracellular mitochondria; F, filtration; HI, healthy individuals; HS, high-speed centrifugation; LP-WGS, low-pass WGS; LS, low-speed centrifugation; mtDNA, mitochondrial DNA; nDNA, nuclear DNA; PE, paired-end; PPw/oPA, plasma preparation without platelet activation; Q-PCR, quantitative PCR; SPP, standard preparation protocol; SPRI, solid-phase reversible immobilization; ssDNA, single-stranded DNA; SSP, ssDNA library preparation; SSP-S, SSP-sequencing; TFAM, mitochondrial transcription factor A; WGS, whole genome sequencing.
