# Supplementary material for: Comparison of the structures and topologies of plasma extracted circulating nuclear and mitochondrial cell-free DNA
Source: Front Genet. 2023 Apr 20;14:1104732. doi: 10.3389/fgene.2023.1104732 (PMC10158822; doi:10.3389/fgene.2023.1104732)
Supplement: Supplementary file 1 [file Table1.docx]

**SUPPLEMENTARY MATERIAL:**

**Supplementary Table 1:** characteristics of the seven healthy individuals whose blood samples were used for the WGS analysis and differential centrifugations.

**Supplementary Table 2:** Number of reads of circulating mitochondrial (cir-mtDNA) and nuclear DNA (cir-nDNA), obtained by low-pass WGS using double-stranded DNA library preparation sequencing (DSP-S) and single-stranded DNA library preparation sequencing (SSP-S) in seven healthy individuals.

**Supplementary Table 3:** Frequencies of mitochondrial circulating DNA fragments < 90 bp (nt), > 200 bp (nt) and ratio (Freq< 90) / (Freq >200) in seven healthy individual samples (A, B) obtained by low-pass WGS using: A, double-stranded DNA library preparation (DSP); B, single-stranded DNA library preparation (SSP)

**Supplementary Table 4:** **Comparison of the concentrations of circulating nuclear DNA (cir-nDNA) and mitochondrial DNA (cir-mtDNA), from blood samples of five healthy individuals prepared by various methods**. Blood samples were obtained respectively (A) from the cirDNA standard plasma preparation (cirDNA-SPP), and (B, C), from the plasma preparation without platelet activation (PPw/oPA). The physical procedures applied after plasma preparation protocols were low speed centrifugation (LS, 1,200 or 2,500 g), filtration, high speed centrifugation (HS, 16,000 g), freezing, or combinations of centrifugation steps of the respective supernatant at 16,000 g, 40,000 g and 200,000 g.

**Supplementary information: detailed method for copy number calculation of circulating nuclear DNA (cir-nDNA) and mitochondrial DNA (cir-mtDNA).**

**Supplementary Table 1**

| **Patient number** | **Age** | **Gender** |
| --- | --- | --- |
| **1** | 25 | M |
| **2** | 59 | M |
| **3** | 46 | M |
| **4** | 47 | M |
| **5** | 23 | F |
| **6** | 31 | F |
| **7** | 30 | M |

**Supplementary Table 2**

|  | **Sample ID** | **Number of cir-mtDNA reads** | **Mean number of cir-mtDNA reads +/- SD** | **Number of cir-nDNA reads** | **Mean number of cir-nDNA reads +/- SD** | **Ratio of cir-mtDNA / cir-nDNA reads** |
| --- | --- | --- | --- | --- | --- | --- |
| **DSP** | 1 | 207 | 79 +/- 60 | 1,611,205 | 1,434,487 +/- 183,186 | 0.006% |
|  | 2 | 79 |  | 1,541,577 |  |  |
|  | 3 | 78 |  | 1,364,840 |  |  |
|  | 4 | 51 |  | 1,079,717 |  |  |
|  | 5 | 24 |  | 1,466,109 |  |  |
|  | 6 | 51 |  | 1,591,437 |  |  |
|  | 7 | 62 |  | 1,386,525 |  |  |
| **SSP** | 1 | 277 | 119 +/- 79 | 1,013,129 | 1,007,070 +/- 204,896 | 0.012% |
|  | 2 | 134 |  | 1,094,997 |  |  |
|  | 3 | 87 |  | 708,192 |  |  |
|  | 4 | 84 |  | 802,664 |  |  |
|  | 5 | 25 |  | 963,701 |  |  |
|  | 6 | 89 |  | 1,299,291 |  |  |
|  | 7 | 140 |  | 1,167,517 |  |  |

**Supplementary Table 3**

| **A** | **Sample ID** | **Frequency < 90 bp (%)** | **Frequency > 200 bp (%)** | **Ratio (Freq<90) / (Freq>200)** | |
| --- | --- | --- | --- | --- | --- |
|  | **1** | 0.97 | 42.03 | 0.02 | |
|  | **2** | 2.53 | 40.51 | 0.06 | |
|  | **3** | 2.56 | 30.77 | 0.08 | |
|  | **4** | 0 | 43.14 | 0 | |
|  | **5** | 0 | 37.50 | 0 | |
|  | **6** | 0 | 37.25 | 0 | |
|  | **7** | 0 | 35.48 | 0 | |
|  | **Mean healthy DSP +/- SD** | **0.9 +/- 1.2** | **38.1 +/- 4.2** | **0.02 +/- 0.03** | |
|  | | | | | |
| **B** | **Sample ID** | **Frequency < 90 nt (%)** | **Frequency > 200 nt (%)** | | **Ratio (Freq<90) / (Freq>200)** |
|  | **1** | 30.0 | 12.64 | | 2.37 |
|  | **2** | 35.1 | 15.67 | | 2.24 |
|  | **3** | 40.2 | 16.09 | | 2.50 |
|  | **4** | 27.4 | 11.90 | | 2.30 |
|  | **5** | 32.0 | 4.00 | | 8.00 |
|  | **6** | 30.3 | 13.48 | | 2.25 |
|  | **7** | 39.3 | 12.14 | | 3.24 |
|  | **Mean healthy SSP +/- SD** | **33.5 +/- 4.9** | **12.3 +/- 4.0** | | **3.3 +/- 2.1** |

**Supplementary Table 4**

| **A** | **Supernatant from the cirDNA-SPP** | **cir-mtDNA** | | | | **cir-nDNA** | | | | **cir-mtDNA/ total cirDNA** |
| --- | --- | --- | --- | --- | --- | --- | --- | --- | --- | --- |
|  |  | **mean** | **mean** |  | **SEM** | **mean** | **mean** |  | **SEM** | **mean** |
|  |  | **copies/mL** | **ng/ml** | **%** | **%** | **copies/mL** | **ng/ml** | **%** | **%** | **%** |
|  | **LS (1,200 g)** | 382,860,000 | 1.2762 | 100.0 |  | 1599 | 5.276 | 100.0 |  | 19.48 |
|  | **LS + filtration** | 4,500,000 | 0.015 | 1.3 | 0.94 | 1373 | 4.532 | 105.8 | 121 | 0.33 |
|  | **LS + HS (16,000 g)** | 1,920,000 | 0.0064 | 0.6 | 0.32 | 2196 | 7.248 | 146.2 | 103 | 0.09 |
|  | **LS + freezing + HS (16,000 g)** | 24,060,000 | 0.0802 | 7.8 | 7.43 | 2810 | 9.272 | 186.8 | 101 | 0.86 |

| **B** | **Supernatant from the PPw/oPA** | **cir-mtDNA** | | | | **cir-nDNA** | | | | **cir-mtDNA/ total cirDNA** |
| --- | --- | --- | --- | --- | --- | --- | --- | --- | --- | --- |
|  |  | **mean** | **mean** |  | **SEM** | **mean** | **mean** |  | **SEM** | **mean** |
|  |  | **copies /mL** | **ng/mL** | **%** | **%** | **copies/mL** | **ng/mL** | **%** | **%** | **%** |
|  | **LS (2,500 g)** | 5,646,000 | 0.0188 | 100.0 |  | 1075 | 3.5483 | 100.0 |  | 0.53 |
|  | **LS (2,500 g) + filtration** | 1,176,000 | 0.0039 | 20.0 | 6.80 | 1063 | 3.5071 | 105.9 | 33 | 0.11 |
|  | **LS (1,200 g) + HS (16,000 g)** | 1,512,000 | 0.0050 | 24.3 | 13.9 | 994 | 3.2794 | 98.1 | 26 | 0.15 |
|  | **LS (1,200 g) + HS + filtration** | 942,000 | 0.0031 | 15.6 | 8.1 | 994 | 3.2810 | 91.4 | 15 | 0.10 |

| **C** | **Differential centrifugations** | **cir-mtDNA** | | | | **cir-nDNA** | | | | **cir-mtDNA / total cirDNA** |
| --- | --- | --- | --- | --- | --- | --- | --- | --- | --- | --- |
|  |  | **mean** | **mean** |  | **SEM** | **mean** | **mean** |  | **SEM** | **mean** |
|  |  | **copies/mL** | **ng/mL** | **%** | **%** | **copies/mL** | **ng/mL** | **%** | **%** | **%** |
|  | **16,000 g** | 3,678,000 | 0.01226 | 100.0 |  | 1001 | 3.305 | 100.0 |  | 0.37 |
|  | **16,000 g + 40,000 g** | 916,800 | 0.003056 | 24.1 | 28.1 | 705 | 2.325 | 69.74 | 53 | 0.13 |
|  | **16,000 g + 40,000 g + 200,000 g** | 113,400 | 0.000378 | 4.2 | 3.3 | 165 | 0.544 | 16.60 | 5 | 0.07 |

**Supplementary information**

**Cir-mtDNA copy number calculation:**

Cir-mtDNA copy number per milliliter of plasma/supernatant was determined with the following calculation:

$$\boldsymbol{Q mito=}\left( \frac{\boldsymbol{C*Na}}{\boldsymbol{2*MW* Lvector}} \right)\boldsymbol{*}\left( \frac{\boldsymbol{V elution}}{\boldsymbol{V plasma/supernatant}} \right)$$

Q_mito_ is the cir-mtDNA copy number per milliliter of plasma/supernatant;

C is the cir-mtDNA mass concentration (g.μL^−1^) determined by Q-PCR targeting the mitochondrial *MT-CO3* gene;

Na is Avogadro’s number (6.02 × 10^23^ molecules per mole);

L_vector_ is the plasmid length (nucleotides);

MW is the molecular weight of one nucleotide (g.mol^−1^);

V_elution_ is the elution volume of cfDNA extract (μL);

and V_plasma ∕ supernatant_ is the volume of plasma or supernatant used for the extraction (mL).

**Cir-nDNA copy number calculation:**

Cir-nDNA copy number per milliliter of plasma/supernatant was determined with the following calculation:

$$\boldsymbol{Q} \boldsymbol{nuclear}\mathbf{=}\left( \frac{\boldsymbol{c}}{\mathbf{3.3}} \right)\mathbf{*}\left( \frac{\mathbf{V elution}}{\mathbf{V plasma/supernatant}} \right)$$

Qnuclear is the cir-nDNA copy number per milliliter;

C is the cir-nDNA concentration (pg.μL^−1^), determined by Q-PCR targeting the nuclear *KRAS* gene sequence;

3.3 pg is the human haploid genome mass;

V_elution_ is the volume of cfDNA extract (μL);

and V_plasma ∕ supernatant_ is the volume of plasma or supernatant used for the extraction (mL).
